# Patterns of genetic diversity of the cryptogenic red alga *Polysiphonia morrowii* (Ceramiales, Rhodophyta) suggest multiple origins of the Atlantic populations

**DOI:** 10.1002/ece3.2135

**Published:** 2016-07-19

**Authors:** Alexandre Geoffroy, Christophe Destombe, Byeongseok Kim, Stéphane Mauger, María Paula Raffo, Myung Sook Kim, Line Le Gall

**Affiliations:** ^1^ UPMC Univ Paris 06UMI 3614Biologie évolutive et écologie des alguesStation Biologique de RoscoffPlace Georges Teissier29682RoscoffFrance; ^2^ CNRSUMI 3614Biologie évolutive et écologie des alguesStation Biologique de Roscoff29682RoscoffFrance; ^3^ Department of BiologyJeju National University66 JejudaehaknoJeju‐si, Jeju‐do690‐756Korea; ^4^ Laboratorio de Algas Marinas BentónicasCentro para el Estudio de Sistemas Marinos (CESIMAR)Centro Nacional Patagónico (CENPAT–CONICET)Bvd. Brown 2915Puerto MadrynU9120ACFChubutArgentina; ^5^ Muséum National d'Histoire Naturelle (MNHN)Institut de Systématique, Biodiversité, ISYEB ‐ UMR 7205 ‐ CNRS, MNHN, UPMCEPHE 57 rue CuvierCP 3975231Paris Cedex 05France

**Keywords:** *cox*1, cryptic species, introduction pathways, *Polysiphonia morrowii*, *rbc*L, red alga

## Abstract

The red alga *Polysiphonia morrowii*, native to the North Pacific (Northeast Asia), has recently been reported worldwide. To determine the origin of the French and Argentine populations of this introduced species, we compared samples from these two areas with samples collected in Korea and at Hakodate, Japan, the type locality of the species. Combined analyses of chloroplastic (*rbc*L) and mitochondrial (*cox*1) DNA revealed that the French and Argentine populations are closely related and differ substantially from the Korean and Japanese populations. The genetic structure of *P. morrowii* populations from South Atlantic and North Atlantic, which showed high haplotype diversity compared with populations from the North Pacific, suggested the occurrence of multiple introduction events from areas outside of the so‐called native regions. Although similar, the French and Argentine populations are not genetically identical. Thus, the genetic structure of these two introduced areas may have been modified by cryptic and recurrent introduction events directly from Asia or from other introduced areas that act as introduction relays. In addition, the large number of private cytoplasmic types identified in the two introduced regions strongly suggests that local populations of *P. morrowii* existed before the recent detection of these invasions. Our results suggest that the most likely scenario is that the source population(s) of the French and Argentine populations was not located only in the North Pacific and/or that *P. morrowii* is a cryptogenic species.

## Introduction

Interoceanic human activities (shipping, aquaculture, fishing) have favored interconnected seas and oceans, enhancing species dispersal and increasing the risk of introduction into coastal marine ecosystems (Carlton and Geller 1993). Coastal invasions are one of the major factors contributing to the erosion of marine biodiversity today (Molnar et al. 2008). A biological invasion consists of the occurrence of a taxon beyond its native range (typically referred to as an alien or nonindigenous species, NIS) that has a negative impact on the environment or on human activities. Generally, pathways for species dispersal remain poorly understood on a global scale (Mack et al. 2000). Tracking the origin of the introduction as well as the colonization pathway is frequently a difficult task and often requires population genetics tools (e.g., Holland 2000; Saltonstall 2002; Estoup and Guillemaud 2010; Rius et al. 2015; Yang et al. 2015). Furthermore, these pathways are sometimes so complex that determining the native and introduced status of some species is almost impossible and these species have been qualified as “cryptogenic species” (Carlton 1996). In particular, introduction of taxa that lack conspicuous characters to distinguish between species that look alike (Knowlton 1993) may go undetected until long after the introduction event.

The advent of molecular systematics considerably facilitated species identification and contributed to the detection of more than 300 invasive species in the marine realm (Molnar et al. 2008). About 14% of the recorded invasive marine species are seaweeds (for a review of introduced seaweeds, see Williams and Smith 2007).

Vectors of introduction reported for seaweeds include hull fouling, ballast water, shellfish farming, aquaculture, scientific research, and fishing gear (Vaz‐Pinto et al. 2014). Among these vectors, shipping and aquaculture have both been incriminated in the dispersal of the edible kelp wakame (*Undaria pinnatifida*) originating from Asia; however, patterns of genetic diversity suggest that shipping is the main vector of recurrent introductions in Australasia, and aquaculture is responsible for the introduction and the spread of the species in Europe (Voisin et al. 2005).

Although the exchange of material for aquaculture purposes is difficult to trace, numerous model approaches have been recently developed to improve predictions of the invasion route with respect to shipping activities (e.g., Seebens et al. 2013; Xu et al. 2014). Shipping routes, as vectors of introduction of marine species, are likely cause of the non‐natural redistribution of algae. Recurrent introductions thus appear to be more likely the rule than the exception. Given that the loss of genetic variation expected on invasive populations (i.e., the invasion paradox) can be counterbalanced by multiple introduction events ensuring invasion success (see Roman and Darling 2007), the presence of bioinvasion highways may explain why successful marine NIS often show populations with high genetic diversity in the introduced range (see for review Rius et al. 2015).

**Figure 1:**
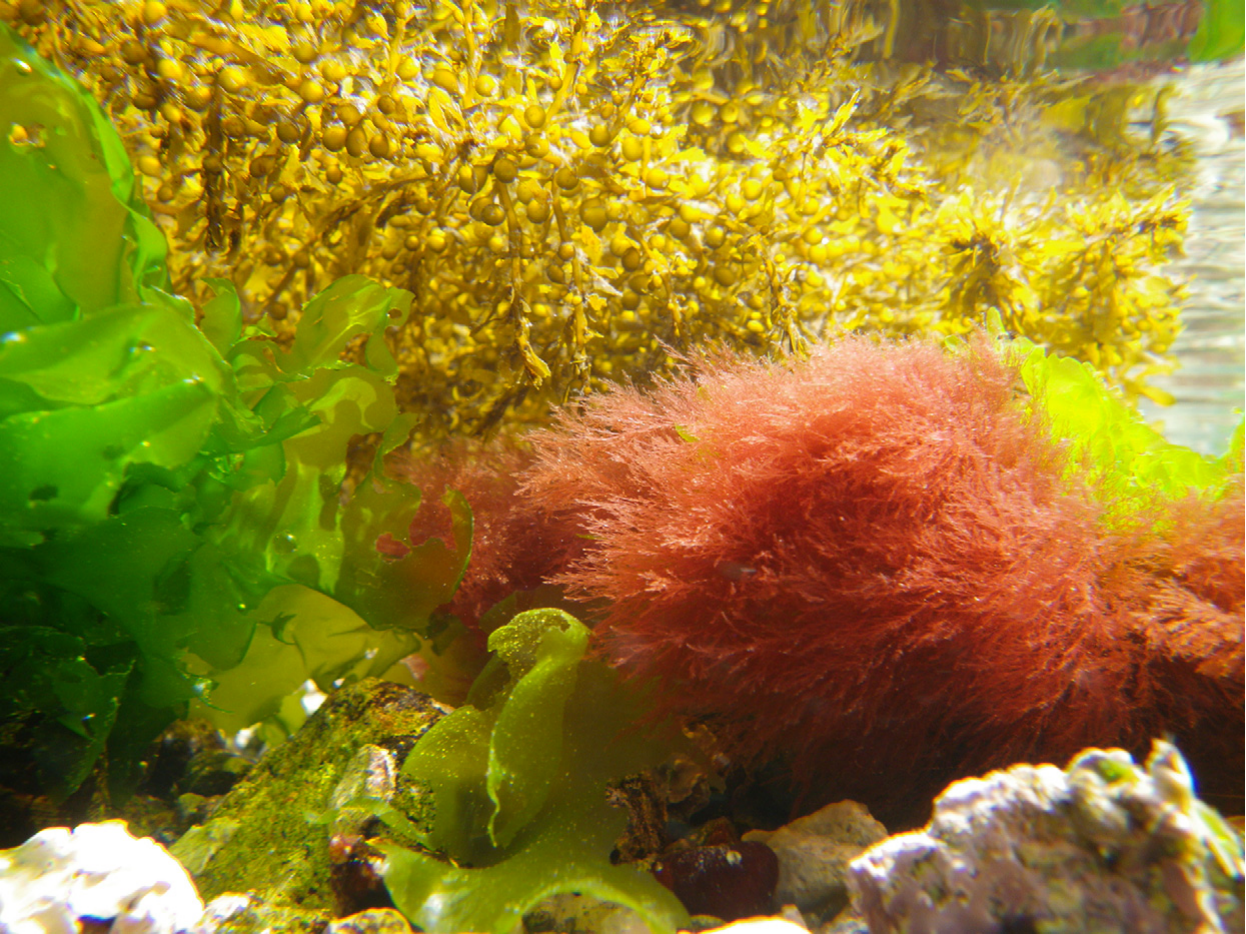
*Polysiphonia morrowii* is a non‐native red alga along Brittany coasts and it forms extensive, dense, and conspicuous patches of individuals in the higher intertidal zones. *P. morrowii* is considered to be native from the North Pacific Ocean and it probably arrived in Europe by human activities. This species was recently identified in Brittany using molecular tools even though it was probably unnoticed for long time due to its morphological similarities with the relative autochthon species, *P. stricta* and *P. atlantica*.

Among the recently reported invasive seaweeds, *Polysiphonia morrowii*, a red alga (Ceramiales, Rhodomelaceae) described by Harvey in 1853 based on the individuals collected in the East Sea at Hokkaido (Hakodate, Hokkaido, Japan), has been reported in various marine ecoregions of the world (Spalding et al. 2007; Thomsen et al. 2016). Its native range is considered to be the temperate North Pacific with records from Japan (Kudo and Masuda 1992), Korea (Kim et al. 1994), China (Segi 1951), and the Russian Far East (Perestenko 1980). The introduction of this species has been recorded in the Mediterranean Sea (Verlaque 2001; Curiel et al. 2002; Erduǧan et al. 2009), the South Pacific Ocean in Chile (Kim et al. 2004) and New Zealand (Mamoozadeh and Freshwater 2012; D'Archino et al. 2013), the North Sea (Maggs and Stegenga 1999), the North Atlantic Ocean in France (Geoffroy et al. 2012) as well as the South Atlantic in Argentina (Croce and Parodi 2014; Raffo et al. 2014). *Polysiphonia morrowii* has been described in Europe as a cryptic introduction based on a DNA barcode approach (Geoffroy et al. 2012; Fig. 1).

The aim of this study was to assess the history of invasion of this species as well as to determine whether the invasion success is associated with several introduction events. To do so, we compared the genetic diversity of *P. morrowii* from Northeast Asia, its putative native range, with that of France and Argentina, two regions of introduction. This comparison was conducted using population genetic approaches based on the mitochondrial and chloroplast markers to characterize and assess the native populations collected from Korea and introduced populations collected in France and Argentina.

## Materials and Methods

### Samples

More than 300 individuals of *P. morrowii* were sampled in three different regions: the North Pacific (Korea and Japan, 168 individuals), the South Atlantic (Argentina, 56 individuals), and the North Atlantic (France, 192 individuals). In addition, 105 specimens sampled in France for a previous study (Geoffroy et al. 2012) were also included in the analyses. As suggested by Muirhead et al. (2008), in order to increase our chance to correctly match introduced individuals to their source population, we performed a sampling design favoring the number of localities over the number of individuals per population. Eleven localities separated by 10–80 km were sampled in Korea (5–10 individuals per site) around Jeju Island (in the Korea Strait) and six (separated by 800 km) along the east and west coast of Korea. The most distant populations were separated by about 1000 km (between Hakodate, Japan and Deoksan, Korea). In contrast, because the ability to correctly resolve the source of an invasion increases with the number of individuals surveyed per introduced population (Muirhead et al. 2008), we increased the number of individuals sampled per population and decreased the number of localities sampled in the North Atlantic. Eight localities were sampled in France, with seven sites 2–450 km apart along the Brittany coast (7–131 specimens per site) and one locality from the Mediterranean Sea (Gulf of Lion) (four specimens). All sites in Brittany were located in the intertidal zone on rocky shores, except one that was located in a marina (Perros‐Guirec). Finally, intertidal rocky shores from three localities sited, between 5 and 30 km apart, were sampled in Nuevo Gulf, Patagonia Argentina (11–29 specimens per site). A fragment of tissue from each sampled individual was preserved in silica gel for molecular analysis. Moreover, at least one specimen per site was pressed and mounted on a herbarium sheet and conserved at the Roscoff Biological Station/French National Museum of Natural History.

### Molecular analyses

DNA was extracted from 5 to 10 mg of dry algal tissue using the Nucleospin^®^ Multi‐96 plant kit (Macherey‐Nagel GmbH and Co. KG, Düren, Germany) according to the manufacturer's protocol. The chloroplastic *rbc*L gene and the mitochondrial *cox*1 gene were amplified using an Eppendorf thermocycler following the protocols described in Guillemin et al. (2008) and Saunders (2005), respectively. *rbc*L gene was amplified with the pair of primers *rbc*L‐F (5′‐CWAAAATGGGATATTGGGAT‐3′) and *rbc*L‐R (5′‐CTATACAYTHGYTGTTGGAGTTTC‐3′). *cox*1 gene was amplified with the pair of primers GazF1 (5′‐TCAACAAATCATAAAGATATTGG‐3′) and GazR1 (5′‐ACTTCTGGATGTCCAAAAAAYCA‐3′). Reaction mixtures (in a total of 25 *μ*L) contained 0.5× PCR buffer (Abgene), 125 *μ*mol/L each dNTP, 1 pmol each primer, 2.5 mmol/L MgCl_2_, 1 U Taq polymerase (Abgene), and 3 *μ*L of DNA (1:25 dilution); PCR cycling included an initial denaturing step at 94°C for 3 min, followed by 35 cycles at 94°C for 45 sec, 50°C for 60 sec, and 72°C for 90 sec with a final elongation step of 72°C for 7 min. The same thermocycler conditions were used for both loci. Finally, PCR products were purified and sequenced by LGC genomics (Berlin, Germany). The sequences were edited and aligned using Codoncode Aligner v. 3.7 (www.codoncode.com).

### Diversity

Partial *rbc*L and *cox*1 sequences were obtained for 521 *Polysiphonia morrowii* individuals including 353 individuals from introduced populations and 168 from its native range. Molecular diversity indices, haplotype diversity (*H*, the probability that two randomly chosen chlorotypes or mitotypes are different) and nucleotide diversity (*π*, the probability that two randomly chosen homologous nucleotide sites are different), were calculated for each sampled location and for each region using Arlequin v 3.11 (Excoffier et al. 2005). To compare haplotype richness (rh) across regions, rarefaction was used to correct for unequal sample sizes using FSTAT 2.9.3 software (Goudet 1995), with *n* = 56 for the chloroplastic and mitochondrial data. Each region was thus considered as a set of 56 individuals. Haplotype richness was recalculated on the individual populations in each region, and significance was computed using a nonparametric permutation test with 2000 permutations. Similar analyses were performed to infer the diversity (1) in France based on *n* = 25, excluding two locations for which sample size was too small (Perros‐Guirec and Mediterranean), (2) in Argentina with *n* = 11, and (3) in Korea with *n* = 7 excluding one location (Seogeondo). To compare haplotype diversities across sampling locations, rarefaction was used to correct for unequal sample sizes (*n* = 10). Haplotype richness estimates were calculated using EstimateS 9.1.0 (Colwell et al. 2012) with each region considered as a single sample. Mean rarefaction curves and the nonparametric estimator (Chao1) were estimated for each gene and cytoplasmic type (the association between mitotype and chlorotype) with 1000 runs of randomization. We extrapolated rarefaction curves with a factor of 1.5 to the sample set.

Phylogenetic relationships among chlorotypes and mitotypes were reconstructed using median‐joining networks using Network software version 4.2.0.1 (Bandelt et al. 1999).

To test for genetic divergence among three regions, populations were grouped according to their geographic location. A hierarchical analysis of molecular variance (AMOVA) was implemented in Arlequin v 3.11 (Excoffier et al. 2005) to analyze the partitioning of genetic variance among and within the three geographic regions. Φ‐statistics were calculated as pairwise differences among locations and their significance was evaluated using a nonparametric permutation test with 10,000 permutations. Moreover, the genetic structure between sampled areas was implemented in GENEPOP web version 4.0.10 (Raymond and Rousset 1995) by calculating an estimate of *F*_ST_ (Weir and Cockerham 1984).

## Results

### Chloroplast diversity

**Figure 2:**
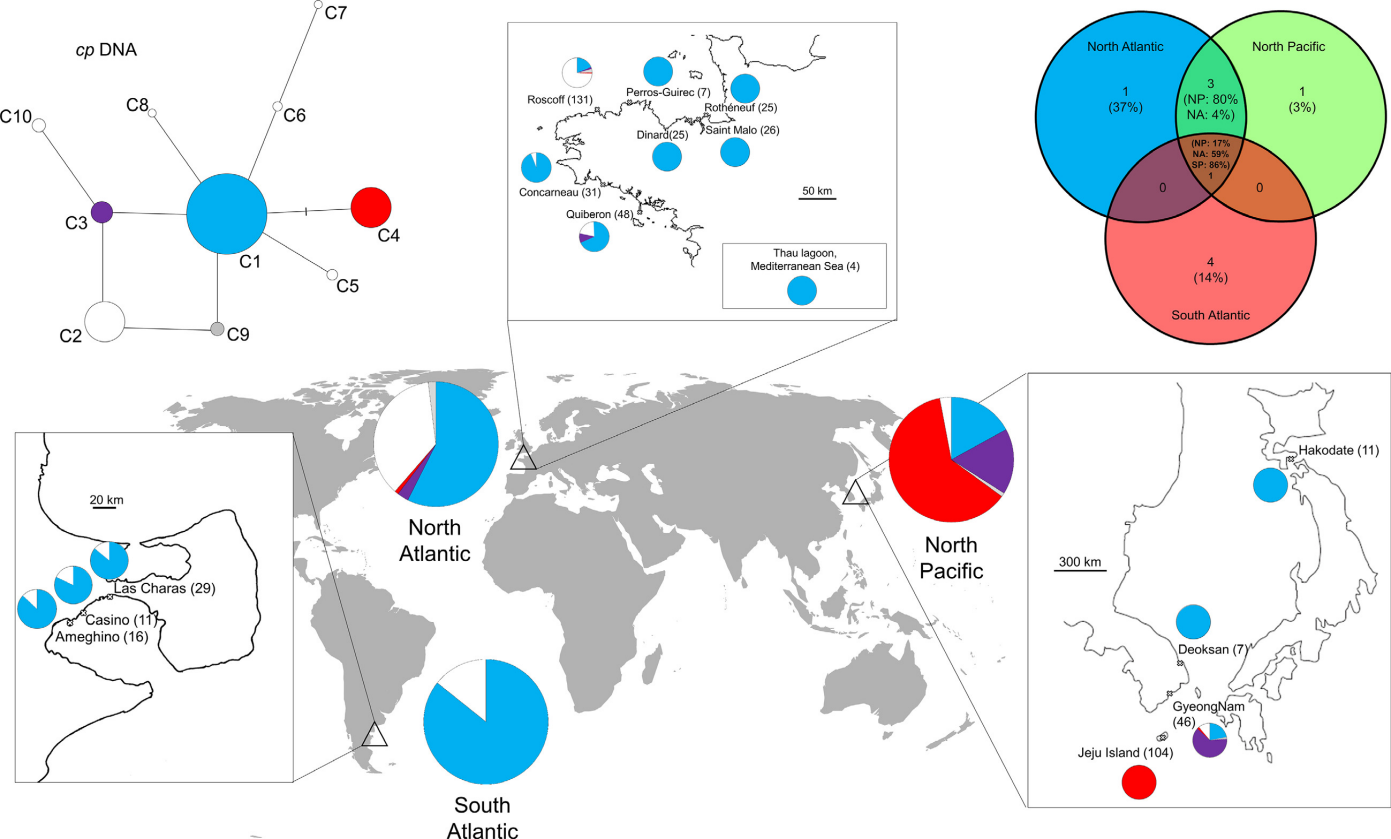
Diversity and the distribution of chlorotypes of *Polysiphonia morrowii* collected in the North Atlantic (*n* = 297), the South Atlantic (*n* = 56), and the North Pacific (*n* = 168). Different colors represent distinct chlorotypes. Private chlorotypes are shown in white. Left, median‐joining network analysis of relationships among of *rbc*L sequences in 521 individuals of *P. morrowii*. Circle surface area is proportional to chlorotype frequency. Lines drawn between chlorotypes represent single mutational steps, and small bars represent additional mutational steps. In the top right, Venn diagram representing chlorotypes shared within the three different areas.

After editing *rbc*L sequences, an alignment of 1225 bp was built on 521 individuals. Ten polymorphic chlorotypes and nine polymorphic sites (0.73%) were observed (GenBank accession number: KP729448–KP729457, Supporting information). Chlorotypes differed by 1–4 bp (Fig. 2). The distribution of these chlorotypes is given in Figure 2. Over the whole dataset, three chlorotypes (C1, C2, and C4) were found at high frequency (>20%) compared with the others that showed a frequency lower than 7% (C3: 6.9%, C5: 0.8%, C6: 0.4%, C7: 0.2%, C8: 0.2%, C9: 1.2%, and C10: 1%). The most frequent chlorotype C1 was observed in 250 individuals (48%) and corresponded to the central chlorotype in the network. The C2 was observed in 110 individuals (21%), and C4 was observed in 106 individuals (20%).

Six chlorotypes (60%) were unique to a single region: C2 was found only in the North Atlantic, C10 was found only in the North Pacific, and four chlorotypes (C5, C6, C7, and C8) were found only in the South Atlantic (Fig. 2). Chlorotype C4 was the most frequent in the North Pacific and it was found only once in the North Atlantic (1%). Chlorotype C1 was the only one present in all three regions. Although it was a frequent chlorotype in the North Atlantic (58%) and the South Atlantic (86%), it was less common in the North Pacific (17%).

### Mitochondrial diversity

**Figure 3:**
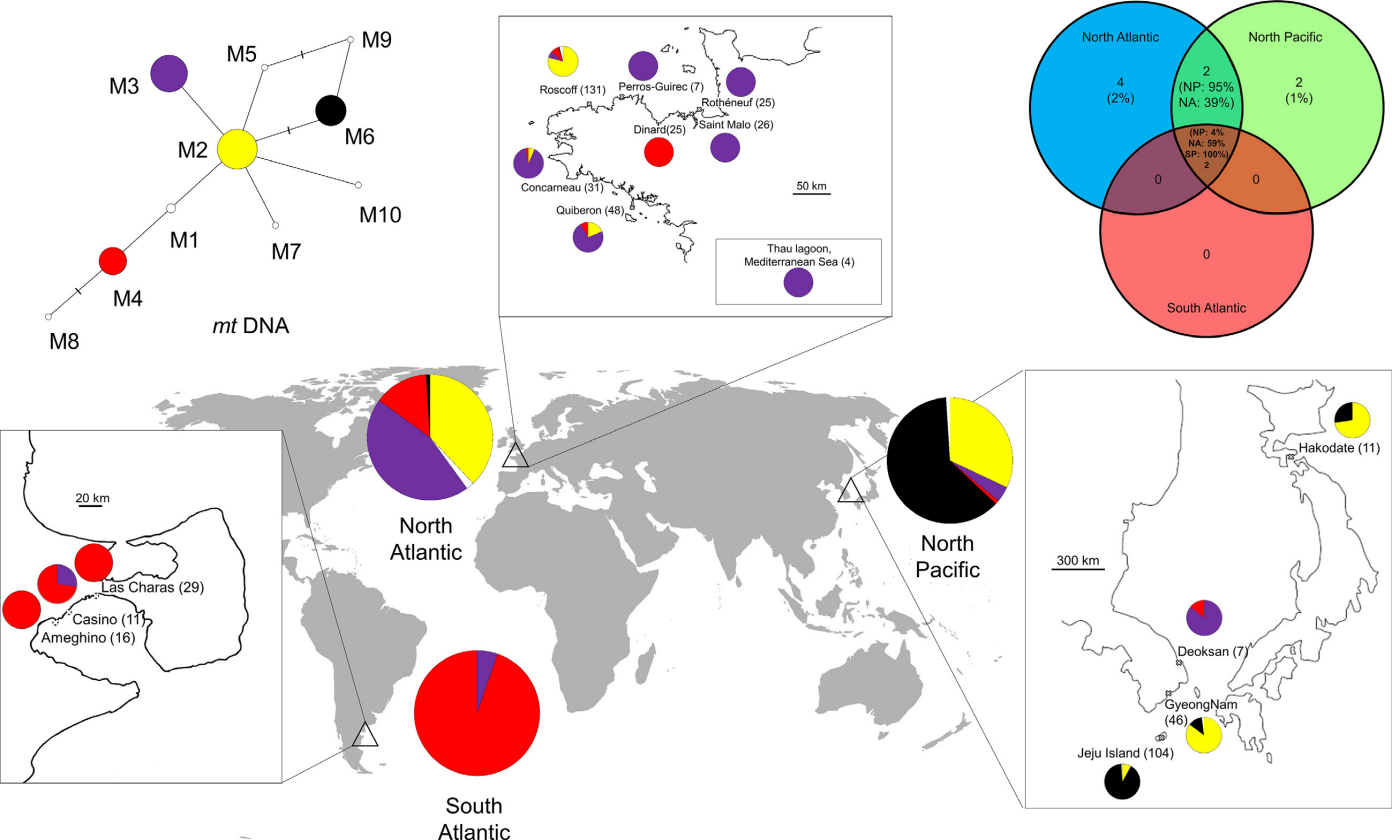
Diversity of *Polysiphonia morrowii* and the distribution of mitotypes collected in the North Atlantic (*n* = 297), the South Atlantic (*n* = 56) and the North Pacific (*n* = 168). Different colors represent distinct mitotypes. Private mitotypes are shown in white. Left, median‐joining network analysis of relationships among of the *cox*1 sequence in 521 individuals of *P. morrowii*. Circle surface area is proportional to mitotype frequency. Lines drawn between mitotypes represent single mutational steps, and small bars represent additional mutational steps. In the top right, Venn diagram representing mitotypes shared within the three different areas.

After editing *cox*1 sequences, an alignment of 559 bp was built on 521 individuals. Ten polymorphic sites (1.8%) defining 10 mitotypes were observed (GenBank accession number: KP729458–KP729467, Supporting information). Pairs of mitotypes were separated by 1–7 bp (Fig. 3). The *cox*1 network revealed four frequent mitotypes (Fig. 3). The most frequent haplotype M2 (32%) was found at the center of the network. The mitotypes M3, M6, and M4 were found at frequencies of 27%, 20%, and 18.5%, respectively. Six mitotypes M1, M5, M7, M8, M9, and M10 were infrequent (between 0.2% and 0.6%). Two mitotypes (M3 and M4) were common to all three regions (South Atlantic, North Atlantic, and North Pacific), and two other mitotypes (M2 and M6) were shared only between the North Atlantic and the North Pacific (Fig. 3). Four mitotypes (50%) were unique to the North Atlantic (M1, M5, M7, and M8) and two mitotypes (1%) were unique to the North Pacific (M9 and M10). The North Pacific and the North Atlantic shared four mitotypes: three (M2, M3, and M4) were being abundant in the North Atlantic, whereas only one (M6) was abundant in the North Pacific.

### Cytoplasmic type diversity

**Figure 4:**
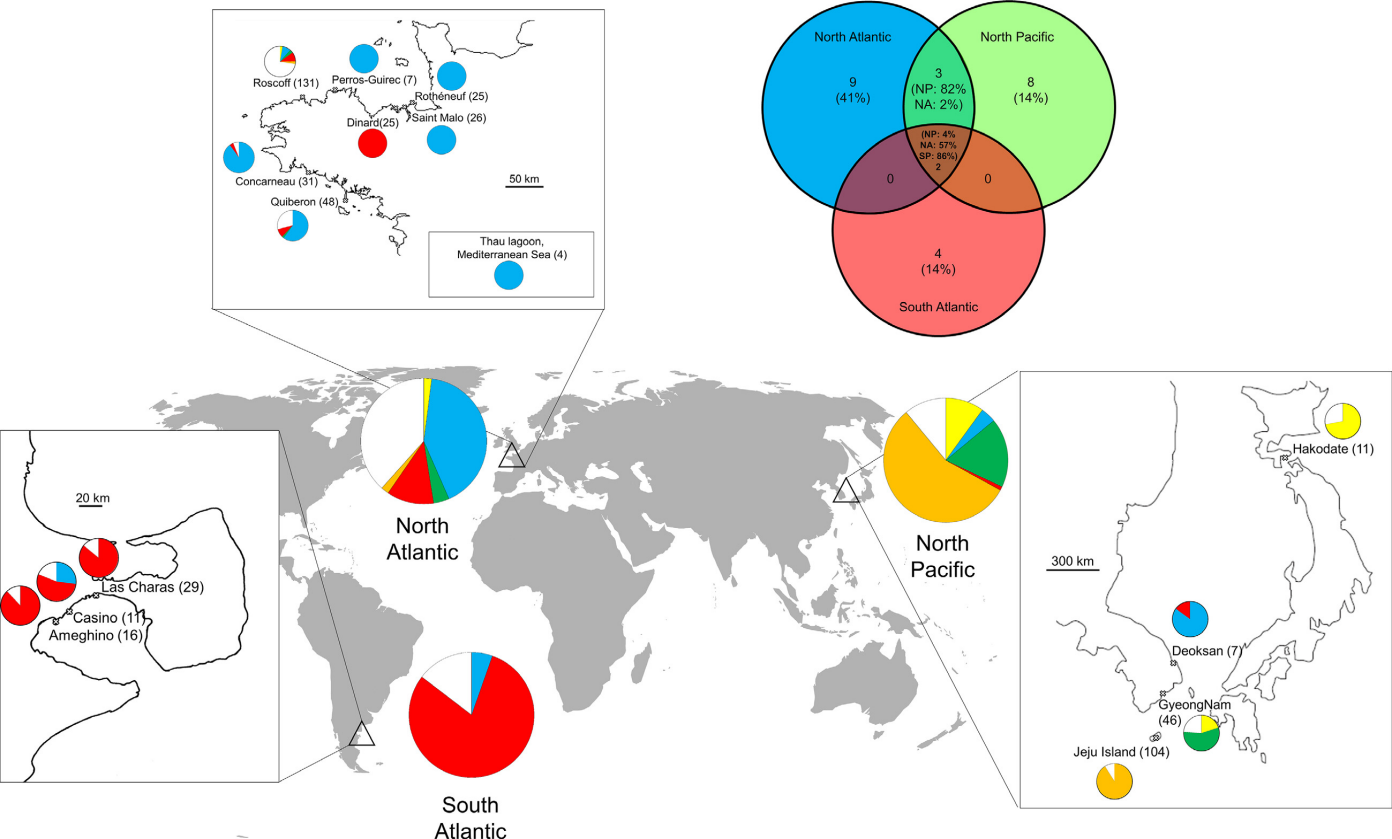
Diversity of *Polysiphonia morrowii* collected in the North Atlantic (*n* = 297), the South Atlantic (*n* = 56), and the North Pacific (*n* = 168) and the distribution of cytoplasmic types. Different colors represent distinct cytoplasmic types, that is, associations between chlorotypes and mitotypes. Private cytoplasmic types are shown in white. In the top right, Venn diagram representing cytoplasmic types shared within the three different areas.

The association between chlorotypes and mitotypes for the 521 individuals is given in Table 1. More than 70% of individuals had cytoplasmic types shared by at least two regions. The cytoplasmic type C1_M3 (26%) was the most frequent and was found in all three regions (South Atlantic, North Atlantic, and North Pacific), whereas the next most frequent cytoplasmic type C2_M2 (20%) was only observed in the North Atlantic (Fig. 4). The cytoplasmic type C4_M6 (91% of the sampled individuals) was mainly observed in the North Pacific in which 11 populations from Jeju Island, Korea, were composed mainly of cytoplasmic type C4_M6 (Fig. 4). In Korea, the Goseong population showed cytoplasmic type C3_M2 and the Deoksan population and four populations from the Gyeongnam Province in the south shared several cytoplasmic types. The Japan population (considered as the native population) showed two cytoplasmic types: C1_M2 also identified in the Gyeongnam Province and in Roscoff population (France) and C1_M6 only present in North Pacific.

**Table 1:** Association between chloroplastic (*rbc*L) and mitochondrial (*cox*1) sequences in *Polysiphonia morrowii* from the North Pacific, North Atlantic, and South Atlantic

*rbc*L	*cox*1									
	M1	M2	M3	M4	M5	M6	M7	M8	M9	M10
C1	3^NA^	18	137	87		4^NP^		1^NA^		
C2		105^NA^	2^NA^	1^NA^	1^NA^		1^NA^			
C3		29	4^NA^			2^NP^			1^NP^	
C4		9^NP^				96^NP^*				1^NP^
C5				4^SA^						
C6				2^SA^						
C7				1^SA^						
C8				1^SA^						
C9		5^NA^				1^NP^				
C10		3^NP^				2^NP^				

The number given for each genetic combination corresponds to the number of individuals bearing this cytoplasmic type.

NP, private cytoplasmic type of North Pacific; NA, private cytoplasmic type of the North Atlantic; SA, private cytoplasmic type of the Southwest Atlantic; NP*, cytoplasmic type found in 98.9% in the North Pacific samples.

Three populations from the South Atlantic showed relatively high genetic diversity, with at least three different cytoplasmic types, and populations from Ameghino and Las Charas featured two additional, unique cytoplasmic types C7_M4 and C8_M4, respectively. In the North Atlantic, population diversity was contrasted among sampled localities. Roscoff, Concarneau, and Quiberon showed greater haplotype diversity (*H* ranged from 0.249 to 0.775) and greater haplotype richness (rh ranged from 1.4 to 2.5) than the other sites (*H* = 0, rh = 1). We identified the same unique cytoplasmic type C1_M3 in Rothéneuf, Saint‐Malo, Perros‐Guirec, and the Mediterranean Sea (Fig. 4). The Dinard population showed only one cytoplasmic type, C1_M4. With 14 cytoplasmic types, Roscoff showed the highest number of cytoplasmic types of all populations (Fig. 4).

**Figure 5:**
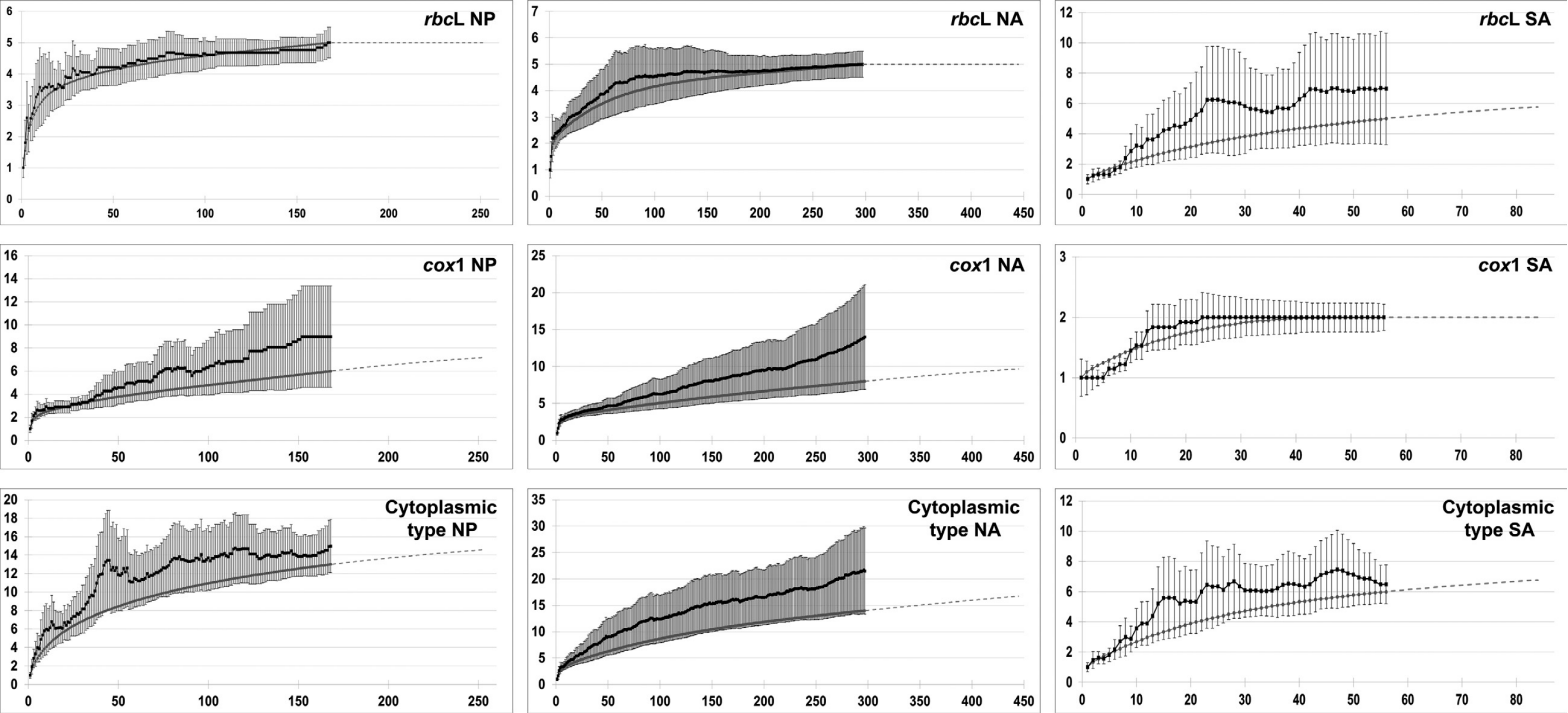
Rarefaction analyses of chlorotype, mitotype, and cytoplasmic type diversity for the three study regions (NP: North Pacific, NA: North Atlantic, and SA: South Atlantic). Reference samples (gray lines), Chao1 mean estimator (black lines) (and standard errors) and extrapolation curves (dashed lines) are shown. Horizontal axis and vertical axis, respectively, correspond to the number of individuals sampled and the number of haplotypes or cytoplasmic types.

Rarefaction analysis of the *rbc*L gene suggested that all chlorotypes present in the native area and in the North Atlantic were sampled (five chlorotypes for each area): Both the sample data and the Chao1 estimate curves leveled off (Fig. 5). However, in the South Atlantic, the rarefaction curve did not reach an asymptote, indicating that sampling effort was insufficient to estimate the genetic diversity adequately. Conversely, for the mitochondrial *cox*1 gene, the rarefaction curve reached an asymptote, indicating that all mitotypes were sampled (2), in the South Atlantic, which was not the case in the other two regions. Finally, the analysis indicated that the diversity of the cytoplasmic types was not described in its entirety for any of the three regions.

### Population structure

Over the whole dataset, the number of chlorotypes was similar to that of mitotypes, regardless of the region: 5, 5, and 5 chlorotypes versus 6, 8, and 2 mitotypes, for the North Pacific, North Atlantic, and South Atlantic regions, respectively (Table 2). The sequence divergences estimated by haplotype diversity (*H*) and nucleotide diversity (*π*) for the chloroplastic and the mitochondrial markers are given in Table 2. The estimates of genetic diversity (*H*) were similar for *rbc*L and *cox*1, regardless of the region considered (Table 2). The values of chloroplast and mitochondrial haplotype richness were not significantly different among regions (nonparametric permutation test, *P*‐value = 0.92). The genetic diversity of the native populations (North Pacific) varied widely among locations. The populations of the Dugok, in GyeongNam Province, Korea, showed relatively high diversity (*H* = 0.69 for *rbc*L and *H* = 0.56 for *cox*1), whereas the populations from locations on Jeju island were much less variable (*H* = 0.00 for *rbc*L and *H* = 0.00–0.56 for *cox*1) (Table 2). Genetic variation in introduced populations (North Atlantic and South Atlantic) also varied considerably. In Brittany, the population established in Quiberon showed relatively high level of variability (*H* = 0.48 for *rbc*L and *H* = 0.43 for *cox*1), whereas the populations of Saint‐Malo showed no variation at all (*H* = 0.00 for *rbc*L and *H* = 0.00 for *cox*1). In introduced regions, a reduction in genetic diversity was observed in the South Atlantic region compared with the North Atlantic region (*H* and *π*, Table 2). Pairwise comparison among regions (AMOVA) revealed that the genetic diversity between the North Atlantic and the South Atlantic was not significantly different for the *rbc*L gene (Table 3) and the majority of variation was significantly partitioned among and within populations, not between regions. Pairwise analyses between the North Pacific and the two Atlantic regions showed that the genetic variation (chloroplastic and mitochondrial) was equally explained by the differentiation between regions and the differentiation between populations within each region. For *rbc*L gene, the lowest genetic differentiation value was observed between the Northern and Southern Atlantic regions, whereas the highest values were observed between native and introduced regions (Table 3). Unlike, for *cox*1 gene, the lowest genetic differentiation values were observed between Pacific and Atlantic Northern regions. Genetic differentiation between pairs of populations (*F*_ST_) is given in supplementary material (Table S1).

**Table 2:** Sampling locations and diversity measures for chloroplastic (*rbc*L) and mitochondrial (*cox*1) genes in *Polysiphonia morrowii*

Location	*n*	*rbc*L				*cox*1			
		nh	rh	*H* (SD)	*π* × 10^3^ (SD)	nh	rh	*H* (SD)	*π* × 10^3^ (SD)
North Pacific (Korea and Japan)	168	5	3.6	0.509 (0.040)	1.068 (0.742)	6	3	0.498 (0.031)	1.863 (1.380)
Deoksan, Gangwondo	7	1	1	0	0	2	1.8	0.286 (0.196)	1.533 (1.398)
Goseong, GyeongNam	7	1	1	0	0	1	1	0	0
Dugok, GyeongNam	9	4	3.3	0.694 (0.147)	1.451 (1.048)	2	2	0.556 (0.090)	1.988 (1.617)
Honghyeon, GyeongNam	11	1	1	0	0	3	2.2	0.345 (0.172)	1.496 (1.297)
Mijori, GyeongNam	8	2	2	0.535 (0.123)	0.437 (0.458)	1	1	0	0
Sachon, GyeongNam	11	2	2	0.436 (0.133)	0.356 (0.388)	2	1.6	0.182 (0.143)	0.651 (0.762)
Ongpo, Jeju	10	1	1	0	0	1	1	0	0
Gimnyeong, Jeju	10	1	1	0	0	1	1	0	0
Hamdeok, Jeju	10	1	1	0	0	1	1	0	0
Seongsan, Jeju	10	1	1	0	0	1	1	0	0
Geumneung, Jeju	10	1	1	0	0	1	1	0	0
Sehwa, Jeju	10	1	1	0	0	1	1	0	0
Seogeondo, Jeju	5	1	1	0	0	1	1	0	0
Sagye, Jeju	10	1	1	0	0	1	1	0	0
Pyoseon, Jeju	10	1	1	0	0	2	1.9	0.355 (0.159)	1.272 (1.174)
Hado, Jeju	9	1	1	0	0	3	2.6	0.555 (0.165)	2.186 (1.733)
Ojori, Jeju	10	1	1	0	0	2	2	0.533 (0.095)	1.908 (1.551)
Hakodate, Hokkaido, Japan	11	1	1	0	0	2	2	0.436 (0.133)	1.561 (1.335)
North Atlantic (France)	297	5	2.8	0.520 (0.017)	0.791 (0.597)	8	3.5	0.631 (0.014)	1.876 (1.383)
Rothéneuf	25	1	1	0	0	1	1	0	0
Saint‐Malo	26	1	1	0	0	1	1	0	0
Dinard	25	1	1	0	0	1	1	0	0
Perros‐Guirec	7	1	1	0	0	1	1	0	0
Roscoff	131	5	2.3	0.405 (0.046)	0.599 (0.496)	8	2.4	0.370 (0.052)	1.103 (0.971)
Concarneau	31	2	1.4	0.124 (0.077)	0.204 (0.263)	3	1.7	0.185 (0.090)	0.554 (0.652)
Quiberon	48	3	2.5	0.483 (0.070)	0.633 (0.522)	3	2.3	0.435 (0.075)	1.280 (1.084)
Mediterranean	4	1	1	0	0	1	1	0	0
South Atlantic (Argentina)	56	5	3.1	0.263 (0.076)	0.253 (0.293)	2	1.7	0.103 (0.054)	0.554 (0.643)
Casino	11	2	1.9	0.327 (0.153)	0.267 (0.327)	2	2	0.436 (0.133)	2.342 (1.781)
Ameghino	16	3	2	0.242 (0.135)	0.293 (0.335)	1	1	0	0
Las Charas	29	4	2	0.258 (0.104)	0.221 (0.276)	1	1	0	0

*n*, Number of individuals per sampling location; nh, number of identified haplotypes; rh, haplotype richness after rarefaction to 56 individuals for regions, and, at the population level, to seven individuals for the North Pacific, 25 individuals for the North Atlantic, and 11 individuals for the South Atlantic; *H*, haplotype diversity (SD, standard deviation), *π* nucleotidic diversity (SD, standard deviation).

**Table 3:** Hierarchical analysis of molecular variance for each marker in pairwise comparisons of regions

Marker	Source of variation	df	Sum of squares	Variance component	% Variance	Φ‐statistics
North Pacific/North Atlantic						
*rbc*L	Among regions	1	102.559	0.40746	39.23	Φ_CT_ = 0.392^a^
	Among populations within regions	24	174.285	0.44711	43.05	Φ_SC_ = 0.708^a^
	Within populations	439	80.803	0.18406	17.72	Φ_ST_ = 0.822^a^
	Total	464	357.647	1.03862		
*cox*1	Among regions	1	105.908	0.43237	42.06	Φ_CT_ = 0.420^a^
	Among populations within regions	24	151.866	0.38651	37.60	Φ_SC_ = 0.648^a^
	Within populations	439	91.818	0.20915	20.34	Φ_ST_ = 0.796^a^
	Total	464	349.591	1.02804		
North Pacific/South Atlantic						
*rbc*L	Among regions	1	34.933	0.30011	32.99	Φ_CT_ = 0.329^a^
	Among populations within regions	19	100.947	0.51461	56.57	Φ_SC_ = 0.844^a^
	Within populations	203	19.285	0.09500	10.44	Φ_ST_ = 0.895^a^
	Total	223	155.165	0.90972		
*cox*1	Among regions	1	107.126	1.20752	71.72	Φ_CT_ = 0.717^a^
	Among populations within regions	19	60.216	0.29467	17.50	Φ_SC_ = 0.619^a^
	Within populations	203	36.814	0.18135	10.77	Φ_ST_ = 0.892^a^
	Total	223	204.156	1.68354		
North Atlantic/South Atlantic						
*rbc*L	Among regions	1	14.783	0.06391	11.15	Φ_CT_ = 0.111
	Among populations within regions	9	74.139	0.28187	49.18	Φ_SC_ = 0.553^a^
	Within populations	342	77.752	0.22735	39.67	Φ_ST_ = 0.603^a^
	Total	352	166.674	0.57312		
*cox*1	Among regions	1	67.251	0.59374	51.20	Φ_CT_ = 0.512^a^
	Among populations within regions	9	95.595	0.36674	31.63	Φ_SC_ = 0.648^a^
	Within populations	342	68.095	0.19911	17.17	Φ_ST_ = 0.828^a^
	Total	352	230.941	1.15959		

a Significance is based on 10,000 permutations: <0.001.

## Discussion

The use of chloroplastic and mitochondrial marker genes has increased in the past decade for the assessment of inter‐ and intraspecific genetic diversity (Sherwood et al. 2010) and to trace the origin of introduced seaweed species (Voisin et al. 2005; Kim et al. 2010; Rueness 2010; Geoffroy et al. 2012; Dijoux et al. 2014). The combined use of both cytoplasmic genomes to trace introduction routes in seaweed is relatively rare (Sherwood et al. 2011). In most Rhodophyta species, cytoplasmic genomes are characterized by clonal reproduction and maternal cotransmission (Zuccarello et al. 1999a, 1999b; Zuccarello and West 2003; Destombe et al. 2010; but see Choi et al. 2008). Therefore, the combination of chloroplastic and mitochondrial markers (cytoplasmic types) can be a powerful marker for tracing origins of introduced populations because both types of markers are transmitted from one generation to the next without recombination (Birky 2001).

In our study, the combination of these two cytoplasmic markers revealed 26 cytoplasmic types, distributed both among and within populations. Species diversity for both markers (*H* and *π*, see Table 2) was similar for the introduced North Atlantic and for the native North Pacific but lower for the South Atlantic, even after accounting for its relatively small sample size. The majority of the Atlantic populations (60%) were polymorphic, with two to five different cytoplasmic types per population. Surprisingly, our results show high genetic structure among Asian populations corresponding to low within‐population genetic diversity and little sharing of cytoplasmic types among the sampled North Pacific populations. For example, populations from Jeju Island, Deoksan, Goseong (Korea), and Hakodate (Japan) did not share any cytoplasmic types, suggesting that populations are genetically isolated. These discrepancies between native and introduced populations suggest that *P. morrowii* was likely introduced in the Atlantic by multiple introduction events from different native populations. However, monomorphism was observed in four introduced populations in Brittany, namely Rothéneuf, Saint‐Malo, Perros‐Guirec, and Dinard, suggesting a severe population bottleneck arising from a single‐event introduction. Although population bottlenecks and founder effects have been reported to be a common characteristic of colonization events leading to depressed genetic diversity in introduced populations compared with the source populations (Tsutsui et al. 2000), recent reviews indicate that the loss of variation is, on average, limited (Dlugosch and Parker 2008; Rius et al. 2015). Recurrent introduction events are frequently cited as a possible explanation of enhanced variation on introduced populations (Roman and Darling 2007).

The South Atlantic and North Atlantic regions showed similar cytoplasmic type structures. Two cytoplasmic types, C1_M3 and C1_M4, present in all three regions, were abundant in the North and South Atlantic, but rare in the North Pacific. The presence of these two cytoplasmic types in all three geographically distant regions is good evidence that *P. morrowii* is a recent introduction in at least two of these regions. We hypothesize that one region served as a stepping stone for the other. Given that the diversity is higher in the North Atlantic, it is likely that this latter region was the intermediate source for the introduction of *P. morrowii* in the South Atlantic.

Various studies of seaweed introductions (e.g., *Caulerpa taxifolia* (Meusnier et al. 2004), *Neosiphonia harveyi* (McIvor et al. 2001), *Gracilaria vermiculophylla* (Kim et al. 2010) have suggested that many widespread introductions may have originated from a particularly successful introduced population (corresponding to a restricted number of genotypes) rather than from genotypes representative of the native range (Lombaert et al. 2010). For example, patterns of genetic diversity suggest that the first introduction of *U. pinnatifida* on the Atlantic coasts originated from a population already established in the Thau lagoon (Mediterranean Sea), where it was accidentally introduced (Voisin et al. 2005). Similarly, in *P. morrowii*, the most frequent cytoplasmic type observed in Brittany (C1_M3) was identical to that detected in Thau lagoon on the French Mediterranean coast (Fig. 4). Thau lagoon has been considered as a “hotspot” for introduced species from the Northwest Pacific due to oyster imports since 1970 (Verlaque 2001) and may have been one of the intermediate sources of introduction via aquaculture in Brittany. Interestingly, this cytoplasmic type (C1_M3) was relatively rare in Asian populations, except in the Deoksan samples. Moreover, the cytoplasmic type (C1_M4) frequently detected in the South Atlantic populations and in Dinard (Brittany) was also observed in Deoksan (Korea). Together, these observations strongly suggest recent introduction events of *P. morrowii* in the Atlantic from the region around Deoksan, Korea.

Aquaculture activities and shipping are considered to be the most important vectors for macroalgal introductions (Molnar et al. 2008). In our study, the observation of *P. morrowii* populations in proximity to mollusk‐farming areas in Brittany (i.e., Roscoff, Concarneau, and Quiberon) suggests that *P. morrowii* may have been accidentally introduced during the deliberate import of Pacific oysters *Crassostrea gigas*. Oyster transport has been shown to be a major vector of recurrent seaweed introduction in Europe (Sjøtun et al. 2008; Boudouresque et al. 2011; Farnham 1980; Mineur et al. 2010). Recently, Manghisi et al. (2010) demonstrated that the red alga *Agardhiella subulata*, endemic to the Atlantic coast of North America, was introduced to Sicily from the Netherlands as a plantlet growing on a *C. gigas* shell. *P. morrowii* is reported as an intertidal species and is found on a large variety of substrata including rocks, wooden piles, ropes, mussels, crabs, and shells, as well as other large algae, such as *S. muticum* and *U. pinnatifida* (Kudo and Masuda 1992; Kim et al. 1994). Therefore, repeated import of mollusks and seaweeds is a likely vector of the spread of *P. morrowii* in France.

Nevertheless, it has been shown that ship‐ballasting practices contribute to the establishment of veritable marine invasion highway (Ricciardi and MacIsaac 2000). A species flow network model recently showed that ports in the Pacific are frequent sources of species that invade South America and Western Europe ports (directly or via a Mediterranean stepping stone) (Xu et al. 2014). Moreover, a study assessing the applicability of the metabarcoding methodology for the detection of organisms in ballast waters, carried out during a cruise from Bremerhaven to Cape Town shows that red algae and, in particular, *Polysiphonia* sp. can survive the 21 days of travel in the ballast (Zaiko et al. 2015). In light of these two studies, ballast waters are a credible vector of spread and/or introduction of *P. morrowii*. Nonetheless, the presence of the private chlorotype C2 found only in Brittany (Roscoff, Concarneau, and Quiberon) and four private chlorotypes C5, C6, C7, and C8 detected only in Argentina contradicts this scenario. There are at least three possible origins of these private cytoplasmic types in Brittany and Argentina. First, the phylogenetic analysis indicated that these cytoplasmic types derive from other Asian populations that were not sampled, requiring additional, extensive sampling to detect them. Second, these chlorotypes may correspond to a previous, older introduction in the North Atlantic during the last two centuries. Third, chlorotypes C2, C5, C6, C7, and C8 are all associated with five different mitotypes, possibly corresponding to native lineages of *P. morrowii* that have gone unnoticed in Brittany (Geoffroy et al. 2012) and in Argentina (Raffo et al. 2014) until this study. The morphological similarity with local species *P. stricta* and *P. atlantica* (Kim and Lee 1996) in the North Atlantic and with *P. abscissa* in the South Atlantic may have obscured previous introductions. Although the alien or native status of *P. morrowii* in the Atlantic is difficult to demonstrate – because it is impossible to date *P. morrowii* introductions – the high observed frequencies of private chlorotypes and cytoplasmic types in the North and South Atlantic (Table 1) strongly suggest that *P. morrowii* was already present before recent introduction events.

Our study, which shows relatively high genetic diversity and structure in the North Atlantic and South Atlantic, suggests recent recurrent introduction events through human activities; however, we cannot determine the specific route of introduction nor when this species was introduced. We therefore conclude that *P. morrowii* is a cryptogenic species as defined by Carlton (1996).

## Data Accessibility

DNA sequences: GenBank accessions KP729448–KP729467 and file in Supporting information created with unique DNA sequences for each *mt*DNA and *cp*DNA haplotype.

## Conflict of Interest

None declared.

## Supporting information


**Table S1.** Pairwise region genetic differentiation (pairwise *F*_ST_ estimates).

**Appendix S1.** Sampling and genetic information for 521 individuals.

**Appendix S2.** Unique DNA sequences for each mtDNA and cpDNA haplotype.

## References

[ece32135-bib-0001] Bandelt, H. J. , P. Forster , and A. Röhl . 1999 Median‐joining networks for inferring intraspecific phylogenies. Mol. Biol. Evol. 16:37–48. 10331250 10.1093/oxfordjournals.molbev.a026036

[ece32135-bib-0002] Birky, C. W. 2001 The inheritance of genes in mitochondria and chloroplasts: laws, mechanisms, and models. Annu. Rev. Genet. 35:125–148. 11700280 10.1146/annurev.genet.35.102401.090231

[ece32135-bib-0003] Boudouresque, C. F. , J. Klein , S. Ruitton , and M. Verlaque (2011) Biological invasion: the Thau Lagoon, a Japanese biological island in the Mediterranean Sea Pp. 151–156 *in* Ceccaldi H.‐J., Dekeyser I., Girault M. and Stora G., eds. Mankind‐Marine Environment Interactions, Proceedings of the 13th French‐Japanese Oceanography Symposium, Springer, Dordrecht, the Netherlands.

[ece32135-bib-0005] Carlton, J. T. , and J. B. Geller . 1993 Ecological roulette: the global transport of nonindigenous marine organisms. Science 261:78–82. 17750551 10.1126/science.261.5117.78

[ece32135-bib-0006] Carlton, J. T. 1996 Biological invasions and cryptogenic species. Ecology 77:1653–1655.

[ece32135-bib-0007] Choi, H. G. , G. T. Kraft , H. S. Kim , M. D. Guiry , and G. W. Saunders . 2008 Phylogenetic relationships among lineages of the Ceramiaceae (Ceramiales, Rhodophyta) based on nuclear small subunit rDNA sequence data. J. Phycol. 44:1033–1048. 27041622 10.1111/j.1529-8817.2008.00554.x

[ece32135-bib-0008] Colwell, R. K. , A. Chao , N. J. Gotelli , Lin, S. Y. , C. X. Mao , R. L. Chazdon , et al. 2012 Models and estimators linking individual‐based and sample‐based rarefaction, extrapolation and comparison of assemblages. J. Plant Ecol. 5:3–21.

[ece32135-bib-0009] Croce, M. E. , and E. R. Parodi . 2014 The Japanese alga *Polysiphonia morrowii* (Rhodomelaceae, Rhodophyta) on the South Atlantic Ocean: first report of an invasive macroalga inhabiting oyster reefs. Helgol. Mar. Res. 68:241–252.

[ece32135-bib-0010] Curiel, D. , G. Bellemo , B. L. Rocca , M. Scattolin , and M. Marzocchi . 2002 First report of *Polysiphonia morrowii* Harvey (Ceramiales, Rhodophyta) in the Mediterranean sea. Bot. Mar. 45:66–70.

[ece32135-bib-0011] D'Archino, R. , K. F. Neill , and W. A. Nelson . 2013 Recognition and distribution of *Polysiphonia morrowii* (Rhodomelaceae, Rhodophyta) in New Zealand. Bot. Mar. 56:41–47.

[ece32135-bib-0012] Destombe, C. , M. Valero , and M. L. Guillemin . 2010 Delineation of two sibling red algal species, *Gracilaria gracilis* and *Gracilaria dura* (gracilariales, rhodophyta), using multiple DNA markers: resurrection of the species *G. dura* previously described in the northern Atlantic 200 years ago. J. Phycol. 46:720–727.

[ece32135-bib-0013] Dijoux, L. , F. Viard , and C. Payri . 2014 The more we search, the more we find: discovery of a new lineage and a new species complex in the genus *Asparagopsis* . PLoS One 9:1–13. 10.1371/journal.pone.0103826 PMC4116237 25076489

[ece32135-bib-0014] Dlugosch, K. M. , and I. M. Parker . 2008 Founding events in species invasions: genetic variation, adaptive evolution, and the role of multiple introductions. Mol. Ecol. 17:431–449. 17908213 10.1111/j.1365-294X.2007.03538.x

[ece32135-bib-0015] Erduǧan, H. , C. Aki , O. Acar , B. Dural , and V. Aysel . 2009 New record for the east Mediterranean, Dardanelles (Turkey) and its distribution: *Polysiphonia morrowii* Harvey (Ceramiales, Rhodophyta). Turk. J. Fish. Aquat. Sci. 9:231–232.

[ece32135-bib-0016] Estoup, A. , and T. Guillemaud . 2010 Reconstructing routes of invasion using genetic data: why, how and so what? Mol. Ecol. 19:4113–4130. 20723048 10.1111/j.1365-294X.2010.04773.x

[ece32135-bib-0017] Excoffier, L. , G. Laval , and S. Schneider . 2005 Arlequin (version 3.0): an integrated software package for population genetics data analysis. Evol. Bioinf. Online 1:47–50. PMC2658868 19325852

[ece32135-bib-0018] Farnham, W. F. 1980 Studies on aliens in the marine flora of southern England Pp. 875–914 *in* Price J. H., Irvine D. E. G. and Farnham W. F., eds. The shore environment, volume 2: ecosystems. Academic Press for Systematics Association, London.

[ece32135-bib-0019] Geoffroy, A. , L. Le Gall , and C. Destombe . 2012 Cryptic introduction of the red alga *Polysiphonia morrowii* Harvey (Rhodomelaceae, Rhodophyta) in the north atlantic ocean highlighted by a DNA barcoding approach. Aquat. Bot. 100:67–71.

[ece32135-bib-0020] Goudet, J. 1995 FSTAT (Version 1.2): a computer program to calculate F‐statistics. J. Hered. 86:485–486.

[ece32135-bib-0021] Guillemin, M. L. , S. A. Akki , T. Givernaud , et al. 2008 Molecular characterisation and development of rapid molecular methods to identify species of Gracilariaceae from the Atlantic coast of Morocco. Aquat. Bot. 89:324–330.

[ece32135-bib-0022] Holland, B. S. 2000 Genetics of marine bioinvasions. Hydrobiologia 420:63–71.

[ece32135-bib-0023] Kim, M.‐S. , and I. K. Lee . 1996 Morphology and reproduction of *Polysiphonia atlantica* Kapraun et J. Norris (Rhodomelaceae, Rhodophyta). J. Plant Biol. (Korea Republic) 39:23–29.

[ece32135-bib-0024] Kim, M. S. , I. K. Lee , and S. M. Boo . 1994 Morphological studies of the red alga *Polysiphonia morrowii* Harvey on the Korean coast. Korean J. Phycol. 9:185–192.

[ece32135-bib-0025] Kim, M. S. , C. Y. Eun , A. Mansilla , and M. B. Sung . 2004 Recent introduction of *Polysiphonia morrowii* (Ceramiales, Rhodophyta) to Punta Arenas, Chile. Bot. Mar. 47:389–394.

[ece32135-bib-0026] Kim, S. Y. , F. Weinberger , and S. M. Boo . 2010 Genetic data hint at a common donor region for invasive Atlantic and Pacific populations of *Gracilaria vermiculophylla* (Gracilariales, Rhodophyta). J. Phycol. 46:1346–1349.

[ece32135-bib-0027] Knowlton, N. 1993 Sibling species in the sea. Annu. Rev. Ecol. Syst. 24:189–216.

[ece32135-bib-0028] Kudo, T. , and M. Masuda . 1992 Taxonomic features of *Polysiphonia morrowii* Harvey (Ceramiales, Rhodophyta). Korean J. Phycol. 7:13–26.

[ece32135-bib-0029] Lombaert, E. , T. Guillemaud , J. M. Cornuet , et al. 2010 Bridgehead effect in the worldwide invasion of the biocontrol harlequin ladybird. PLoS One 5:e9743. 20305822 10.1371/journal.pone.0009743 PMC2840033

[ece32135-bib-0030] Mack, R. N. , D. Simberloff , W. M. Lonsdale , et al. 2000 Biotic invasions: causes, epidemiology, global consequences and control. Ecol. Appl. 10:689–710.

[ece32135-bib-0031] Maggs, C. A. , and H. Stegenga . 1999 Red algal exotics on North Sea coasts. Helgolander Meeresunters 52:243–258.

[ece32135-bib-0032] Mamoozadeh, N. R. , and D. W. Freshwater . 2012 *Polysiphonia* sensu lato (Ceramiales, Florideophyceae) species of Caribbean Panama including *Polysiphonia lobophoralis* sp. nov. and *Polysiphonia nuda* sp. nov. Bot. Mar. 55:317–347.

[ece32135-bib-0033] Manghisi, A. , M. Morabito , C. Bertuccio , et al. 2010 Is routine DNA barcoding an efficient tool to reveal introductions of alien macroalgae? A case study of *Agardhiella subulata* (Solieriaceae, Rhodophyta) in Cape Peloro lagoon (Sicily, Italy). Cryptogam. Algol. 31:423–433.

[ece32135-bib-0034] McIvor, L. , C. A. Maggs , J. Provan , and M. J. Stanhope . 2001 *rbc*L sequences reveal multiple cryptic introductions of the Japanese red alga *Polysiphonia harveyi* . Mol. Ecol. 10:911–919. 11348500 10.1046/j.1365-294x.2001.01240.x

[ece32135-bib-0035] Meusnier, I. , M. Valero , J. L. Olsen , and W. T. Stam . 2004 Analysis of rDNA ITS1 indels in *Caulerpa taxifolia* (Chlorophyta) supports a derived, incipient species status for the invasive strain. Eur. J. Phycol. 39:83–92.

[ece32135-bib-0036] Mineur, F. , O. De Clerck , A. Le Roux , C. Maggs , and C.A. Maggs M Verlaque . 2010 *Polyopes lancifolius* (Halymeniales, Rhodophyta), a new component of the Japanese marine flora introduced to Europe. Phycologia 49:86–96.

[ece32135-bib-0037] Molnar, J. L. , R. L. Gamboa , C. Revenga , and M. D. Spalding . 2008 Assessing the global threat of invasive species to marine biodiversity. Front. Ecol. Environ. 6:485–492.

[ece32135-bib-0038] Muirhead, J. R. , D. K. Gray , D. W. Kelly , et al. 2008 Identifying the source of species invasions: sampling intensity vs. genetic diversity. Mol. Ecol. 17:1020–1035. 18261046 10.1111/j.1365-294X.2008.03669.x

[ece32135-bib-0039] Perestenko, L. P. 1980 Algae of Peter the Great Bay. Nauka, Leningrad.

[ece32135-bib-0040] Raffo, M. P. , A. Geoffroy , C. Destombe , and E. Schwindt . 2014 First record of the invasive red alga *Polysiphonia morrowii* Harvey (Rhodomelaceae, Rhodophyta) on the Patagonian shores of the Southwestern Atlantic. Bot. Mar. 57:21–26.

[ece32135-bib-0041] Raymond, M. , and F. Rousset . 1995 GENEPOP (version 1.2): population genetics software for exact tests and ecumenicism. J. Hered. 86:248–249.

[ece32135-bib-0042] Ricciardi, A. , and H. J. MacIsaac . 2000 Recent mass invasion of the North American Great Lakes by Ponto‐Caspian species. Trends Ecol. Evol. 15:62–65. 10652557 10.1016/s0169-5347(99)01745-0

[ece32135-bib-0043] Rius, M. , X. Turon , G. Bernardi , F. A. M. Volckaert , and F. Viard . 2015 Marine invasion genetics: from spatio‐temporal patterns to evolutionary outcomes. Biol. Invasions 17:869–885.

[ece32135-bib-0044] Roman, J. , and J. A. Darling . 2007 Paradox lost: genetic diversity and the success of aquatic invasions. Trends Ecol. Evol. 22:454–464. 17673331 10.1016/j.tree.2007.07.002

[ece32135-bib-0045] Rueness, J. 2010 DNA barcoding of select freshwater and marine red algae (Rhodophyta). Cryptogam. Algol. 31:377–386.

[ece32135-bib-0046] Saltonstall, K. 2002 Cryptic invasion by a non‐native genotype of the common reed, *Phragmites australis*, into North America. Proc. Natl Acad. Sci. USA 99:2445–2449. 11854535 10.1073/pnas.032477999 PMC122384

[ece32135-bib-0047] Saunders, G. W. 2005 Applying DNA barcoding to red macroalgae: a preliminary appraisal holds promise for future applications. Philos. Trans. R. Soc. Lond. B Biol. Sci. 360:1879–1888. 16214745 10.1098/rstb.2005.1719 PMC1609223

[ece32135-bib-0048] Seebens, H. , M. T. Gastner , and B. Blasius . 2013 The risk of marine bioinvasion caused by global shipping. Ecol. Lett. 16:782–790. 23611311 10.1111/ele.12111

[ece32135-bib-0049] Segi, T. 1951 Systematic study of the genus *Polysiphonia* from Japan and its vicinity. J. Facul. Fish., Prefectural University of Mie 1:169–272.

[ece32135-bib-0050] Sherwood, A. R. , T. Sauvage , A. Kurihara , K. Y. Conklin , and G. G. Presting . 2010 A comparative analysis of COI, LSU and UPA marker data for the Hawaiian florideophyte Rhodophyta: implications for DNA barcoding of red algae. Cryptogam. Algol. 31:451–465.

[ece32135-bib-0051] Sherwood, A. R. , A. Kurihara , and K. Y. Conklin . 2011 Molecular diversity of Amansieae (Ceramiales, Rhodophyta) from the Hawaiian Islands: a multi‐marker assessment reveals high diversity within *Amansia glomerata* . Phycol. Res. 59:16–23.

[ece32135-bib-0052] Sjøtun, K. , V. Husa , and V. Peña . 2008 Present distribution and possible vectors of introductions of the alga *Heterosiphonia japonica* (Ceramiales, Rhodophyta) in Europe. Aquat. Invasions 3:377–394.

[ece32135-bib-0053] Spalding, M. D. , H. E. Fox , G. R. Allen , et al. 2007 Marine ecoregions of the world: a bioregionalization of coastal and shelf areas. Bioscience 57:573–583.

[ece32135-bib-0054] Thomsen, M. S. , T. Wernberg , P. M. South , and D. R. Schiel (2016) Non‐native seaweeds drive changes in marine coastal communities around the world Pp. 147–185 *in* Hu Z.‐M. and Fraser C., eds. Seaweed Phylogeography. Springer, Dordrecht, the Netherlands.

[ece32135-bib-0055] Tsutsui, N. D. , A. V. Suarez , D. A. Holway , and T. J. Case . 2000 Reduced genetic variation and the success of an invasive species. Proc. Natl Acad. Sci. USA 97:5948–5953. 10811892 10.1073/pnas.100110397 PMC18539

[ece32135-bib-0056] Vaz‐Pinto, F. , B. Martínez , C. Olabarria , and F. Arenas . 2014 Neighbourhood competition in coexisting species: the native *Cystoseira humilis* vs the invasive *Sargassum muticum* . J. Exp. Mar. Biol. Ecol. 454:32–41.

[ece32135-bib-0057] Verlaque, M. 2001 Checklist of the macroalgae of Thau Lagoon (Hérault, France), a hot spot of marine species introduction in Europe. Oceanol. Acta 24:29–49.

[ece32135-bib-0058] Voisin, M. , C. R. Engel , and F. Viard . 2005 Differential shuffling of native genetic diversity across introduced regions in a brown alga: aquaculture vs. maritime traffic effects. Proc. Natl Acad. Sci. USA 102:5432–5437. 15802466 10.1073/pnas.0501754102 PMC556235

[ece32135-bib-0059] Weir, B. S. , and C. C. Cockerham . 1984 Estimating F‐statistics for the analysis of population structure. Evolution 38:1358–1370. 10.1111/j.1558-5646.1984.tb05657.x 28563791

[ece32135-bib-0060] Williams, S. L. , and J. E. Smith . 2007 A global review of the distribution, taxonomy, and impacts of introduced seaweeds. Annu. Rev. Ecol. Evol. Syst. 38:327–359.

[ece32135-bib-0061] Xu, J. , T. Wickramarathne , E. Grey , et al. 2014 Patterns of ship‐borne species spread : a clustering approach for risk assessment and management of non‐indigenous species spread. preprint arXiv :1401.5407.

[ece32135-bib-0062] Yang, M. Y. , E. C. Macaya , and M. S. Kim . 2015 Molecular evidence for verifying the distribution of *Chondracanthus chamissoi* and *C. teedei* (Gigartinaceae, Rhodophyta). Bot. Mar. 58:103–113.

[ece32135-bib-0063] Zaiko, A. , J. L. Martinez , J. Schmidt‐Petersen , et al. 2015 Metabarcoding approach for the ballast water surveillance – an advantageous solution or an awkward challenge? Mar. Pollut. Bull. 92:25–34. 25627196 10.1016/j.marpolbul.2015.01.008

[ece32135-bib-0064] Zuccarello, G. C. , and J. A. West . 2003 Multiple cryptic species: molecular diversity and reproductive isolation in the *Bostrychia radicans*/*B. moritziana* complex (Rhodomelaceae, Rhodophyta) with focus on North American isolates. J. Phycol. 39:948–959.

[ece32135-bib-0065] Zuccarello, G. C. , G. Burger , J. A. West , and R. J. King . 1999a A mitochondrial marker for red algal intraspecific relationships. Mol. Ecol. 8:1443–1447. 10564449 10.1046/j.1365-294x.1999.00710.x

[ece32135-bib-0066] Zuccarello, G. C. , J. A. West , U. Karsten , and R. J. King . 1999b Molecular relationships within *Bostrychia tenuissima* (Rhodomelaceae, Rhodophyta). Phycol. Res. 47:81–85.

