# Supplementary material for: Patterns of genetic diversity of the cryptogenic red alga Polysiphonia morrowii (Ceramiales, Rhodophyta) suggest multiple origins of the Atlantic populations
Source: Ecol Evol. 2016 Jul 19;6(16):5635–47. doi: 10.1002/ece3.2135 (PMC4983580; doi:10.1002/ece3.2135)
Supplement: Supplementary file 1 — Table S1. Pairwise region genetic differentiation (pairwise F ST estimates). [file ECE3-6-5635-s001.docx]

**Table S1**: Pairwise region genetic differentiation (pairwise F_ST_ estimates). The name (NP–NA-SA) refer to the region sampled. All F_ST_ values were significant (P < 0.001). In the bottom left-hand corner and in the top right-hand corner, pairwise F_ST_ for *rbc*L gene and *cox*1 gene, respectively.

|  | NP | NA | SA |
| --- | --- | --- | --- |
|  |  | *cox*1 | |
| NP | — | 0.48 | 0.74 |
| NA | 0.46 | — | 0.60 |
| SA | 0.43 | 0.26 | — |
|  | *rbc*L | |  |
